# The major risk factor for depression in the Chinese middle-aged and elderly population: A cross-sectional study

**DOI:** 10.3389/fpsyt.2022.986389

**Published:** 2022-11-10

**Authors:** Xiaolin Ni, Huabin Su, Yuan Lv, Rongqiao Li, Chen Chen, Di Zhang, Qing Chen, Shenqi Zhang, Ze Yang, Liang Sun, Qi Zhou, Xiaoquan Zhu, Danni Gao, Sihang Fang, Caiyou Hu, Guofang Pang, Huiping Yuan

**Affiliations:** ^1^The Key Laboratory of Geriatrics, Beijing Institute of Geriatrics, Institute of Geriatric Medicine, Chinese Academy of Medical Sciences, Beijing Hospital/National Center of Gerontology of National Health Commission, Beijing, China; ^2^Jiangbin Hospital of Guangxi Zhuang Autonomous Region, Nanning, China; ^3^School of Population Medicine and Public Health, Chinese Academy of Medical Sciences/Peking Union Medical College, Beijing, China; ^4^Department of Joint and Sports Medicine, Zaozhuang Municipal Hospital Affiliated to Jining Medical University, Shandong, China

**Keywords:** epidemiology, cross-sectional study, depression, major risk factor, chronic pain

## Abstract

**Background:**

The number of patients suffering from depression is continuously increasing in China. Demographic characteristics, physical health levels, and individual lifestyles/healthy behaviors are associated with the severity of depression. However, the major risk factor for depression remains unclear.

**Materials and methods:**

In this investigation, 16,512 patients were screened using the CHARLS (China Health and Retirement Longitudinal Study) database after being determined to be eligible based on the inclusion criteria. Depressive symptoms were evaluated through the CESD-10 (10-item Center for Epidemiological Studies Depression Scale). Consequently, various models were developed based on potential predictive factors, employing stepwise LR (Logistic Regression)/RF (Random Forests) models to examine the influence and weighting of candidate factors that affect depression.

**Results:**

Gender, residential address location, changes in health status following last interview, physical disabilities, chronic pain, childhood health status, ADL (activity of daily living), and social activity were all revealed to be independent risk factors for depression (*p* < 0.05) in this study. Depression has a synergic effect (across chronic pain and age groups). In comparison to other factors, RF results showed that chronic pain had a stronger impact on depression.

**Conclusion:**

This preliminary study reveals that chronic pain is a major risk factor for depression.

## Introduction

Depression is estimated to affect 322 million individuals worldwide, or 4.4% of the global population (1). The incidence of depression cases in China reached 90 million in 2020, making it the second-leading cause of human mortality, after cancer, with incidence numbers for depression cases in China reaching 90 million (2).

Depression is a prevalent chronic condition in the majority of the world’s population; it can impair normal functioning, cause depressive thoughts, and have a negative impact on the quality of life. Furthermore, individuals with severe depressive disorder have a higher risk of developing cardiovascular diseases and obtaining inadequate treatments, as well as a higher risk of morbidity and mortality (3, 4). The major risk for depression is determined by both genes and environmental factors (5). Among them, estimations of depression’s heritability based on twin studies are only about 37% (6), which is significantly less than the impact of environmental factors. Epidemiological studies have demonstrated that physical health, the lifestyle of childhood, and various stress events in life increase the incidence of depression in the middle-aged and elderly population (7–9). Due to the lack of nationally representative epidemiological data (including demographic profiling, health status and functioning, lifestyle behaviors, etc.) on depressive disorders in China (10), the main risk factors for depression in the Chinese middle-aged and elderly population are still unclear, much less identifying modifiable risk factors for depression to strategically prevent and intervene depressive disorders.

Most Chinese depression studies in recent years have been based on the CHARLS (CHARLS, China Health and Retirement Longitudinal Study), a large-scale epidemiological survey of nationwide middle-aged and older individuals that have been included in the depression scale since 2015. These studies separately examined the association between specific risk factors (e.g., chronic diseases, obesity, and physical activity) and depressive disorders, but a comprehensive, systematic analysis of the main risk factors for depression among middle-aged and elderly people in China is still lacking (11–13). Thus, major risk factors for depression were thoroughly and systematically investigated using cross-sectional data from CHARLS to identify feasible and efficient methods for reducing the incidence of depressive disorders.

## Subjects, materials, and methods

### Patients

**FIGURE 1:**
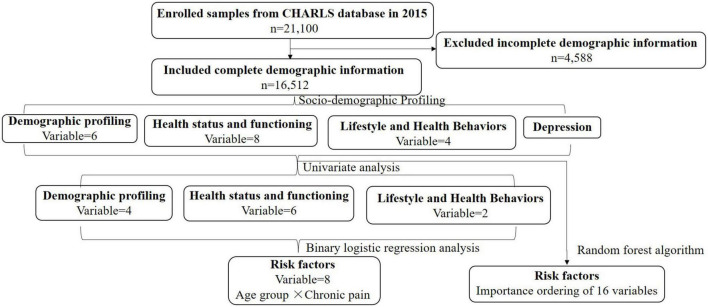
The flowchart of the consecutive analysis steps.

A total of 16,512 participants with complete demographic information were incorporated from 21,100 cases in the wave three surveys of CHARLS. Participants ranged in age from 20 to 102 years old. A flow chart of the consecutive analysis steps is depicted in Figure 1 (Supplementary materials and Supplementary methods. Subjects).

### Demographic profiling

Age, gender, residential address, location, birth address, and marital status were included among the demographic characteristics. Detailed classification criteria for these characteristics can be found in the demographic profiling section of Supplementary materials and Supplementary methods.

### Health status and functioning

The direct inquiry was used to examine self-assessment of the state of health including the present state of health, changes in health status following last interview, physical disabilities, the loss of teeth, chronic pain, other medical diseases or conditions, and health status in childhood (Supplementary materials and Supplementary methods. Health status and functioning).

### Activity of daily living evaluations

The actual physical function of the participants was assessed by ADL scales in this study (14). Participants were separated into three groups based on their overall ADL scores : NFI Group (NFI, No Functional Impairments), MFI Group (MFI, Mild Functional Impairments), and FI Group (FI, Functional Impairments) (15) (Supplementary materials and Supplementary methods. ADL evaluations).

### Healthy/lifestyle behaviors

The direct inquiry was used to evaluate the healthy/lifestyle behaviors such as sporting activities, social activity, smoking, and frequency of drinking (Supplementary materials and Supplementary methods. Healthy/lifestyle behaviors).

### Depressive symptom manifestations

The depressive symptom manifestations of the participants were assessed by the CESD-10 (CESD-10, 10-item Center for Epidemiological Studies Depression Scale) (16). Based on their CESD-10 scores, the participants were classified into three groups: D group (D, depression), DS group (DS, depressive symptoms), and NDS group (NDS, no depressive symptoms) (17) (Supplementary materials and Supplementary methods. Depressive symptom manifestations).

### Statistical analyses

SPSS 26.0^®^ (SPSS Inc.™, USA) was utilized to analyze data statistics, chi-square tests, and binary logistic regression. All *p*-values were two-sided, and *p* < 0.05 was considered statistically significant. The R package caret was used to implement the RF algorithm, which measures the risk factors that cause depression and ranks them by importance. Figure 1 depicts a flowchart of the consecutive analysis steps (Supplementary materials and Supplementary methods. Statistical analyses).

## Results

### Socio-demographic profiling

A total of 16,512 participants were included in the sample, including 8,024 men (48.59%) and 8,488 women (51.41%). According to new guidelines developed by the World Health Organization of the United Nations for the classification of age phases (18), participants in this study were divided into 908 (5.5%) young individuals (20–44 years old), 8,419 (50.99%) middle-aged individuals (45–59 years old), 6,219 (37.66%) younger-old individuals (60–74 years old), 949 (5.75%) older people (75–89 years old), and 17 (0.10%) longevity (above 90 years old). Approximately 98.48% of participants lived at home. The majority of participants (70.21%) resided in a village. Nearly 50% of participants’ birth address was identical to their permanent address. Approximately >80% were married, with a spouse present or cohabiting.

Over 50% of individuals had health conditions that may be considered fair. Following the previous interview, the health status of over 50% of participants remained the same. Only 1,778 (10.17%) people experienced physical impairments, while 663 (4.02%) had lost all of their teeth. In addition, 4,703 (28.48%) participants were frequently affected by chronic pain, 1,259 (7.62%) participants suffered from other medical diseases or conditions, 1,985 (12.02%) participants self-reported having an excellent health status during childhood, whereas the remaining participants, this was either outstanding (6,738; 40.81%), good (3,123; 18.91%), fair 3,656 (22.14%), or poor (1,010; 6.12%). The post-ADL examination revealed that 9,678 individuals (58.61%) had no functional impairments, 5,459 participants (33.06%) had mild functional impairments, and 1,375 individuals (8.33%) had functional impairments. Following post-classification based on a total depression score, 6,143 (37.20%) were assigned to the D group, 8,152 (49.37%) were assigned to the DS group, and 2,217 (13.43%) were assigned to the NDS group.

In terms of lifestyle, 8,194 participants (49.62%) engaged in at least 10 min of continuous physical activity, 7,123 individuals (43.14%) participated in social activities, and 6,586 individuals (39.89%) smoked, and 10,414 individuals (63.07%) did not use alcohol. Table 1 shows specific socio-demographic information for each record.

**TABLE 1:** Sociodemographic characteristics description.

Variables	Num (feq.%)
**Demographic backgrounds**	
Age (years old)	
20–44 (young)	908 (5.50)
45–59 (middle-aged)	8,419 (50.99)
60–74 (young, older man)	6,219 (37.66)
75–89 (the aged)	949 (5.75)
90–110 (longevity)	17 (0.10)
Gender	
Male	8,024 (48.59)
Female	8,488 (51.41)
Type of residential address	
Family housing	16,261 (98.48)
Other	251 (1.52)
Location of residential address	
Main city zone	2,408 (14.58)
Combination-zone (urban-rural)	830 (5.03)
Town/city-center	925 (5.60)
ZhenXiang area	756 (4.58)
Village	11,593 (70.21)
Born address	
Permanent address	7,709 (46.69)
Separate village/neighborhood within permanent address county/city/district	5,743 (34.78)
Other county/city/district	3,060 (18.53)
Marital status	
Married with spouse present/Cohabitated	13,805 (83.61)
Married though temporarily apart due to employment	992 (6.01)
Separated/divorced	195 (1.18)
Widowed	1,416 (8.58)
Never married	104 (0.63)
**Health status and functioning**	
State of health	
Very good	2,122 (12.85)
Good	2,023 (12.25)
Fair	8,453 (51.19)
Poor	3,461 (20.96)
Very poor	453 (2.74)
Changes in health status following last interview	
Better	1,571 (9.51)
About the same	8,188 (49.59)
Worse	6,753 (40.90)
Physical disabilities	
No	14,734 (89.23)
Yes	1,778 (10.77)
The loss of teeth	
Yes	663 (4.02)
No	15,849 (95.98)
Chronic pain	
Yes	4,703 (28.48)
No	11,809 (71.52)
Other medical diseases or conditions	
Yes	1,259 (7.62)
No	15,253 (92.38)
Childhood health status	
Excellent	1,985 (12.02)
Very good	6,738 (40.81)
Good	3,123 (18.91)
Fair	3,656 (22.14)
Poor	1,010 (6.12)
ADL	
No functional impairments (NFI group)	9,678 (58.61)
Mild functional impairments (MFI group)	5,459 (33.06)
Functional impairments (FI group)	1,375 (8.33)
**Lifestyle and health behaviors**	
Sporting activities	
Yes	8,194 (49.62)
No	8,318 (50.38)
Social activity	
Yes	7,123 (43.14)
No	9,389 (56.86)
Smoking	
No	9,926 (60.11)
Yes	6,586 (39.89)
Frequency of drinking	
Drink more than once a month	4,599 (27.85)
Drink but less than once a month	1,499 (9.08)
Do not drink	10,414 (63.07)
Depression	
No depressive symptoms (NDS group)	2,217 (13.43)
Depressive symptoms (DS group)	8,152 (49.37)
Depressive (D group)	6,143 (37.20)

Num, number; freq, frequency.

### Associated factors for depression

Except for age, the variables in this study were all categorical. After the post-normality test, the age did not follow a normal distribution. According to the findings of the ROC curve for follow-up analysis, the study divided age into two groups, with ≤59 and >59 as categorical variables. In contrast to the variance inflation factor, which was significantly less than 10, the examination of collinearity revealed that tolerance was significantly greater than 0.1. There was consequently no collinearity between the independent variables. Univariate analysis revealed a significant difference (*p* < 0.05) between the NDS group and the combined D and DS groups in age, gender, place of residence, place of birth, state of health, changes in health status since the last interview, physical disabilities, chronic pain, childhood health status, ADL, social activity, and smoking (Table 2).

**TABLE 2:** Univariate analysis of independent variables on depressive status.

	Depression		*χ^2^*	*P*-value
Variables	NDS group (*n*/%)	DS+D group (*n*/%)		
**Demographic backgrounds**				
Age group				
Less than 59 years old	1,107 (49.93)	8,220 (57.50)	44.751	2.24E-11
More than 59 years old	1,110 (50.07)	6,075 (42.50)		
Gender				
Male	1,292 (58.28)	6,732 (47.09)	96.098	1.09E-22
Female	925 (41.72)	7,563 (52.91)		
Type of residential address				
Family housing	2,185 (49.28)	14,076 (49.23)	0.101	0.751
Other	32 (0.72)	219 (0.77)		
Location of residential address				
Main city zone	310 (6.99)	2,098 (7.34)	11.846	1.85E-02
Combination-zone (urban-rural)	101 (2.28)	729 (2.55)		
Town/city-center	104 (2.35)	821 (2.87)		
ZhenXiang area	84 (1.89)	672 (2.35)		
Village	1,618 (36.49)	9,975 (34.89)		
Born address				
Permanent address	1,140 (51.42)	6,569 (45.95)	25.883	2.40E-06
Separate village/neighborhood within permanent address county/city/district	677 (30.54)	5,066 (35.44)		
Other county/city/district	400 (18.04)	2,660 (18.61)		
Marital status				
Married with spouse present/cohabitated	1,881 (84.84)	11,924 (83.41)	5.882	0.208
Married though temporarily apart due to employment	129 (5.82)	863 (6.04)		
Separated/divorced	19 (0.85)	176 (1.23)		
Widowed	171 (7.72)	1,245 (8.71)		
Never married	17 (0.77)	87 (0.61)		
**Health status and functioning**				
State of health				
Very good	317 (14.30)	1,805 (12.63)	90.711	9.30E-19
Good	307 (13.85)	1,716 (12.01)		
Fair	1,244 (56.11)	7,209 (50.43)		
Poor	314 (14.16)	3,147 (22.01)		
Very poor	35 (1.58)	418 (2.92)		
Changes in health status following last interview				
Better	235 (10.60)	1,336 (9.35)	68.198	1.55E-15
About the same	1,253 (56.52)	6,935 (48.51)		
Worse	729 (32.88)	6,024 (42.14)		
Physical disabilities				
No	2,041 (92.06)	12,693 (88.79)	21.334	3.86E-06
Yes	176 (7.94)	1,602 (11.21)		
Loss of teeth				
Yes	92 (4.15)	571 (3.99)	0.12	0.729
No	2,125 (95.85)	13,724 (96.01)		
Chronic pain				
Yes	311 (14.03)	4,392 (30.72)	262.657	4.52E-59
No	1,906 (85.97)	9,903 (69.28)		
**Other medical diseases or conditions**				
Yes	148 (6.68)	1,111 (7.77)	3.275	0.07
No	2,069 (93.32)	13,184 (92.23)		
Childhood health status				
Excellent	218 (9.83)	1,767 (12.36)	27.638	1.48E-05
Very good	876 (39.51)	5,862 (41.01)		
Good	463 (20.88)	2,660 (18.61)		
Fair	546 (24.63)	3,110 (21.76)		
Poor	114 (5.15)	896 (6.26)		
ADL				
No functional impairments (NFI group)	1,528 (68.92)	8,150 (57.01)	118.191	2.16E-26
Mild functional impairments (MFI group)	578 (26.07)	4,881 (34.14)		
Functional impairments (FI group)	111 (5.01)	1,264 (8.85)		
**Lifestyle and health behaviors**				
Sporting activities				
Yes	1,135 (51.20)	7,059 (49.38)	2.528	0.112
No	1,082 (48.80)	7,236 (50.62)		
Social activity				
Yes	1,122 (50.61)	6,001 (41.98)	58.265	2.29E-14
No	1,095 (49.39)	8,294 (58.02)		
Smoking				
No	1,208 (54.49)	8,718 (60.99)	33.803	6.10E-09
Yes	1,009 (45.51)	5,577 (39.01)		
Frequency of drinking				
Drink more than once a month	651 (29.36)	3,948 (27.62)	3.238	0.198
Drink but less than once a month	190 (8.57)	1,309 (9.16)		
Do not drink	1,376 (62.07)	9,038 (63.22)		

Num, number; %, frequency.

### Logistic regression analysis for depression

Further logistic regression analysis revealed eight independent risk factors for depression among all 12 variables, including gender, residential address location, changes in health status following the last interview, physical disabilities, chronic pain, childhood health status, ADL, and social activity (*p* < 0.05) (Table 3). Notably, individuals above the age of 59 who did not have chronic pain were protected from depression, and the results of these datasets also revealed a synergic effect between chronic pain and age group.

**TABLE 3:** Binary logistic regression analysis of related factors of depressive symptoms.

Variables		B	SE	Wald	*P*-value	OR	95% CI
Gender	Male						
	Female	0.317	0.048	43.539	4.155E-11	1.373	1.250–1.509
Location of residential address	Main city zone			13.763	0.008		
	Combination-zone (urban-rural)	0.023	0.124	0.036	0.850	1.024	0.803–1.306
	Town/city-center	0.053	0.122	0.190	0.663	1.055	0.830–1.341
	ZhenXiang area	0.078	0.133	0.348	0.555	1.081	0.834–1.403
	Village	–0.168	0.068	6.019	0.014	0.845	0.739–0.967
Changes in health status following last interview	Better			9.837	0.007		
	About the same	0.036	0.079	0.212	0.645	1.037	0.889–1.210
	Worse	0.189	0.083	5.174	0.023	1.208	1.027–1.422
Physical disabilities	No						
	Yes	0.205	0.086	5.656	0.017	1.228	1.037–1.454
Chronic pain	Yes						
	No	–0.623	0.075	69.833	6.453E-17	0.536	0.464–0.621
Childhood health status	Excellent			21.085	3.046E-04		
	Very good	–0.162	0.082	3.921	0.048	0.851	0.725–0.998
	Good	–0.345	0.089	14.878	1.147E-04	0.709	0.595–0.844
	Fair	–0.290	0.087	11.052	0.001	0.748	0.631–0.888
	Poor	–0.088	0.125	0.492	0.483	0.916	0.717–1.171
ADL	NFI group			28.206	7.502E-07		
	MFI group	0.261	0.057	21.228	4.077E-06	1.298	1.162–1.451
	FI group	0.410	0.111	13.580	2.286E-04	1.507	1.212–1.875
Social activity	Yes						
	No	0.373	0.047	61.846	3.715E-15	1.452	1.323–1.593
Age group × Chronic pain		–0.405	0.052	60.268	8.277E-15	0.667	0.602–0.739
Constant		2.249	0.138	265.396	1.144E-59	9.474	

### Feature importance analysis

**FIGURE 2:**
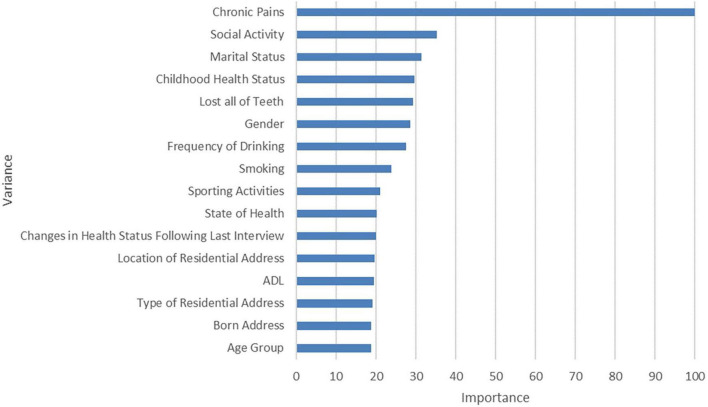
Permutation importance of features in random forest model.

Figure 2 displays the characteristics of the RF model for predicting the result of depression in the order of permutation importance. The *y*-axis displays the predictive variables, while the *x*-axis displays the feature important scores. The predictive variables were scored from high to low. Regarding interpersonal communications, the “chronic pain” variable scored the highest in this model, followed by “social activity” and “marital status.” Regarding this dataset results, RF outperforms LR with an AU-ROC score of 70.0%, whereas LR had an AU-ROC score of 65.5% (Supplementary Figure 1).

## Discussion

Depression is a highly prevalent condition that severely limits psychosocial function and, as a result, reduces life quality (19), contributing to the majority of global health burdens (20). Depression is expected to be the greatest worldwide health burden by 2,030, according to the WHO (19). Working-age adults in China have a high risk of developing depression, which has different degrees of negative effects on their health and longevity. In addition, the onset and severity of depression symptoms tend to increase with age, which is extremely undesirable for the current trend of the Chinese population aging (13). Therefore, it is urgent to explore the main risk factors affecting the occurrence of depression in China, especially in middle-aged and elderly people.

### Depression and basic characteristics of the individual

Depression is a condition that can be influenced by individual differences (21). According to studies, the prevalence of depression is twice as high in women as in men (22). The results of this study also indicated that women are at risk factors for developing depression (*p* < 0.01, OR = 1.373) (Table 3). This could be related to the fact that males and females have distinct biological patterns. The findings revealed that women have higher levels of inflammatory, neurotrophic, and serotonergic markers than men, which may be the biological basis for sex differences in depression (22). Due to the influence of gender and age, marital status has also emerged as one of the risk factors for depression (23). This could also explain why the marital status was not found to be an independent risk factor for depression in the findings of this study. Moreover, the residence is a significant influence on depression. Compared to living in a village and having a permanent address, city life is associated with negative mental health outcomes due to work stress, loss of communication with friends and family in the neighborhood, overcrowding, heavy traffic, and lack of green spaces (24).

### Depression and health status and functioning

From a cultural perspective, physical health was given preferential treatment over mental health throughout China (25). Changes in health status following last interview, physical disabilities, health status in childhood, chronic pain, and ADL as defined by physical health were also found to be independent risk factors for depression in this study (*p* < 0.05) (Table 3). Among them, chronic pain is more significantly related to depression than other variables. Studies conducted in clinical settings demonstrated that chronic pain is a stressful event or condition that frequently promotes depressive disorders, with strong depressive symptoms present in roughly 85% of chronic pain cases. Depression symptom presentation is more prevalent among older patients with chronic pain than among older patients without chronic pain (26). In addition, the prognosis for patients with depression caused by chronic pain was worse than for those with chronic pain alone. Exploring its biological molecular mechanism, it was found that inflammatory signals and monoamine neurotransmitters such as 5-HT (5-HT, serotonin), DA (DA, dopamine), NE (NE, norepinephrine), and glutamate were the key molecules for chronic pain to induce depression (27).

A substantial positive correlation between physical health multimorbidity and depression was discovered in 42 countries, with notably high odds ratios in China, Laos, Ethiopia, the Philippines, and Malaysia, according to a comprehensive international investigation (28). Another CHARLS-based study indicated that individuals with chronic diseases or multimorbidity are more likely to be depressed in the elderly Chinese population, indicating the importance of assessing depressive symptoms in patients who exhibit poor physical performance (11). Other investigations have found a correlation between childhood obesity and an increased incidence of depression in old age (29). In addition to the psychological impact of life’s inconveniences, adults with physical disabilities have fewer opportunities for positive communication with the outside world, which contributes to their greater risk of depression (30). Similarly, CHARLS-based studies demonstrated that social support moderates the influence of disability on depression symptoms (31).

### Depression and healthy/lifestyle behaviors

There is currently a large and validated research base showing lifestyle behaviors as an important cause of depression. These behaviors may be particularly essential in the development and progression of depression (32). For example, good social relationships can play a protective role against the onset of depressive symptoms, particularly in late-life depression (33), which is consistent with the findings of this study in both LR and RF. Physical activity has also been shown to considerably lower the risk of depression and to have antidepressant effects. Physical activity has been shown to influence the onset and progression of depression by altering neuroplasticity, inflammation, oxidative stress, the endocrine system, self-esteem, social support, and self-efficacy (34). Regarding smoking and alcohol use in this study, numerous studies have found a positive relationship between smoking/excessive alcohol consumption and the onset and development of depression (35, 36). Therefore, healthy activities are not only advantageous for maintaining physical health, but they can also have a direct impact on mental health. Through early lifestyle interventions, depression can be prevented or delayed in its onset and progression.

### Strengths and limitation

The primary risk factors for depression in middle-aged and elderly people in China were thoroughly and systematically examined in this study. As a result, according to recent research findings, the chance of developing depression will be significantly reduced if physical health (particularly for the treatment of chronic pain) and lifestyle improvements are identified and implemented promptly and effectively. In the meantime, both LR and RF were used to account for possible overall weighting of the main risk factors (25). The findings of the two models also showed excellent consistency. This study may investigate potential relationships between chronic pain and depression.

In fact, in addition to the influencing factors of depression covered in this study, other factors such as type of tea consumption (37), sleep quality (38), and spirituality/religion (39) are also included. Recent research has demonstrated that religion can be beneficial for modifying lifestyle and enhancing psychological health (40). Harvard University,^1^ Duke University,^2^ and Adventist Religion and Health Study^3^ have carried out research work related to religion. However, due to the lack of relevant data in the CHARLS, this study was not included in the analysis, and it is anticipated that this part will be conducted in the future.

Additionally, depression may be described in a variety of ways utilizing various assessment methods. The ability to compare prevalence estimates from multiple studies employing different depression definitions is limited. Moreover, self-reported health conditions and lifestyle choices may have contributed to memory bias in this study. Even though the AU-ROC scores for the sensitivity analysis were all lower, it demonstrated that chronic pain had a substantial impact on depression using the LR/RF model, which could be related to recall bias.

## Conclusion

In conclusion, this study focused on how physical health and lifestyle affect psychological depression in the Chinese community before identifying that chronic pain had a substantial influence on depression. The comparison analysis of the LR and RF results further supported the consistency of the relationship between chronic pain and depression. Future studies will concentrate on looking for such follow-up data, with an emphasis on the association between depression and chronic pain.

## Data availability statement

The datasets presented in this study can be found in online repositories. The names of the repository/repositories and accession number(s) can be found below: The datasets generated for this study can be found in the China Health and Retirement Longitudinal Study (CHARLS) online datasets (https://charls.charlsdata.com/pages/Data/2015-charls-wave4/zh-cn.html).

## Ethics statement

Our protocols followed the Declaration of Helsinki and received approval from the Ethical Board at Beijing Hospital, Ministry of Health (2019BJYYEC-118-02). Written informed consent to participate in this study was provided by the participants’ legal guardian/next of kin.

## Author contributions

XN and HS contributed to the conception and design of the study and drafted the manuscript. YL, RL, CC, DZ, and QC interpreted the data. ZY, LS, QZ, XZ, DG, SF, and SZ analyzed the data and helped revise the manuscript. CH, GP, and HY helped revise the manuscript. All authors read and approved the final manuscript.

## Footnotes

1
https://cgvh.harvard.edu/national-consortium-psychosocial-stress-spirituality-and-health-0

2
https://spiritualityandhealth.duke.edu/

3
https://publichealth.llu.edu/sites/publichealth.llu.edu/files/docs/sph-ahs-overview.pdf

## Abbreviations

CHARLS: the China Health and Retirement Longitudinal Study
CESD-10: the 10-item Center for Epidemiological Studies Depression Scale
LR: logistic regression
RF: Random Forests
ADL: activity of daily living
D: depression group
DS: depressive symptoms
NDS: the no depressive symptoms
AUC: the area under the curve
NFI group: No Functional Impairments
MFI: Mild Functional Impairments
FI: Functional Impairments
5-HT: serotonin
DA: dopamine
NE: norepinephrine.
