# Supplementary material for: The major risk factor for depression in the Chinese middle-aged and elderly population: A cross-sectional study
Source: Front Psychiatry. 2022 Nov 10;13:986389. doi: 10.3389/fpsyt.2022.986389 (PMC9691648; doi:10.3389/fpsyt.2022.986389)
Supplement: Supplementary file 1 [file Data_Sheet_1.doc]

Supplementary Section for:

**The Major Risk Factor for Depression in the Chinese Middle-aged and Elderly Population: A Cross-Sectional Study**

Xiaolin Ni^1,†^, Huabin Su^2,†^, Yuan Lv^2^, Rongqiao Li^2^, Chen chen^3^, Di Zhang^2^, Qing Chen^2^, Shenqi Zhang^4^, Ze Yang^1^, Liang Sun^1^, Qi Zhou^1^, Xiaoquan Zhu^1^, Danni Gao^1^, Sihang Fang^1^, Caiyou Hu^2,*^, Guofang Pang^2,*^ Huiping Yuan^1,*^

*^1^ The Key Laboratory of Geriatrics, Beijing Institute of Geriatrics, Institute of Geriatric Medicine, Chinese Academy of Medical Sciences, Beijing Hospital/National Center of Gerontology of National Health Commission, Beijing, 100730, P.R.China.*

*^2^ Jiangbin Hospital, Guangxi Zhuang Autonomous Region, Nanning, 530021, P.R.China.*

*^3^* *School of Population Medicine and Public Health, Chinese Academy of Medical Sciences/Peking Union Medical College, No.31, Beijige-3, Dongcheng District, Beijing , 100730, China.*

*^4^ Department of Joint and Sports Medicine, Zaozhuang Muncipal Hospital Affliated to Jining Medical University, Shandong, 277100, China.*

***Corresponding Author:**

Huiping Yuan, yuanhuiping@126.com. Caiyou Hu, cyhu.hua@163.com. Guofang Pang, zeyang1007@126.com. These authors contributed equally to this work and share corresponding authorship.

Xiaolin Ni^1,†^ and Huabin Su^2,†^ : These authors contributed equally to this work and share first authorship.

*Supplementary Section1:* *Supplementary materials and methods.*

**Patients**

The CHARLS is based on a longitudinal survey of Chinese citizens and their socioeconomic and health status evaluations. In addition to previously studied survey topics, a third nationwide survey was undertaken (July – October 2015) with a response rate of 21,100 participants and evaluations for depression-based symptom presentations and Activity of Daily Living (ADL). After obtaining approval, this study examined CHARLS online data sets (December 2021). In line with investigational aims, the current study evaluated participants for the following data: age, gender, type of residential address, location of residential address, birth address, and marital status. Additionally, health-related criteria were: state of health, changes in health status following the last interview, physical disabilities, loss of teeth, chronic pain, other medical conditions, childhood health status, and ADL. Healthy lifestyle/behavior-related criteria included: daily practicing of sports (10-minute session), social activities, smoking, and frequency of drinking. In addition, individuals were required to complete the scoring protocol for the 10-item Center for Epidemiological Studies Depression Scale (CESD-10). For this third survey, 16,512 participants were selected from 21,100 cases based on these inclusion criteria. 4,588 samples with missing data were excluded from this study.

**Demographic profiling**

These characteristics included age, gender, residential address, location, birth address, and marital status. Young individuals (20-44 years old), middle-aged individuals (45-59 years old), younger-old individuals (60-74 years old), older people (75-89 years old), and longevity (≥90 years old) comprised the five age stages established by the United Nations World Health Organization for the classification of age stages (Medina-Solis et al., 2008). Gender was categorized as male or female. The type of residential address was categorized as family housing and other. Location of residential address was identified as main-city-zone, combination-zone (urban-rural), town/city-center, Zhenxiang area, and village. Birth/born address was categorized identically to a permanent address, separate village/neighborhood within permanent address county/city/district, and other county/city/district. Marital status was categorized as married with spouse present/cohabiting, married though temporarily apart due to employment, separated/divorced, widowed, and never married.

**Health status and functioning**

Self-assessment of state of health was measured through the following questions:

1. “Would you say your health is excellent, very good, good, fair, or poor?”
2. “Compared with your health when we talked with you last interview, would you say that your health is better now, about the same, or worse?”
3. “Do you have one for following disabilities”
4. “Have you lost all of your teeth?”
5. “Are you often troubled with any body pains?”
6. “Are there any other medical diseases or conditions that are important to your health now that we have not talked about?”
7. “How would you evaluate your health during childhood, up to and including age 15? Excellent, very good, good, fair, poor?”

The chronic illness states were determined by asking: “Have you been diagnosed with conditions listed below by a doctor?”. Such conditions included hypertension; dyslipidemia; diabetes or hyperglycemia, cancer/malignancies; chronic pulmonary/hepatic/cardiac/renal/cerebrovascular-accident/gastrointestinal conditions, emotional/anxiety, psychiatric conditions, together with memory-related disease, arthritis/rheumatism, and asthma. Experts in such medical fields independently evaluated individual medical issues.

**ADL** **evaluations**

The ADL scale is used in this study to evaluate physical function. It was a 14-item scale to describe the participants’ fundamental and operational ADL (Feng et al., 2021). Individual answers were subdivided within four levels, with responding options being: 1= “No, I do not have any difficulty”, 2 = “I have difficulty but still can do it”, 3 = “Yes, I have difficulty and need help”, and 4 = “I cannot do it”. Increased scores showed reduced ability concerning elderly ADL. Disability was determined as No Functional Impairments (NFI Group), Mild Functional Impairments (MFI Group), and Functional Impairments (FI Group) based on the total scores (Li et al., 2016). The ADL scaling was used for study in China and other countries with good robustness and validity (Chan et al., 2009). The 14 items were further subdivided into five categories: grooming/personal care (dressing, bathing, eating, getting into or out of bed, toileting, and controlling urination and defecation), transportation/mobility (walking and climbing stairs), indoor activities (household chores, cooking, shopping, and phone calls), money-management (bill paying, asset management), and health/medical care (self-administration of medications) (Dennis et al., 2009).

**Healthy/lifestyle behaviors**

The question “ During a usual week, did you do any sports for at least 10 minutes continuously?” was used to determine whether or not someone engaged in sports. Followed by direct, affirmative, or negative response, as necessary.

Social activities were determined by asking: “Have you done any of these activities in the last month?”. Replies included 12 options: 1) interacted with friends; 2) Played Ma-jong, played chess, played cards, or went to a community club; 3) Provided help to family, friends, or neighbors who do not live with you, and so on. The initial 11 options (depicting varying social activities) were combined as “1) Yes” with the final option (“12) None of these”) as “2) No”.

The following questions were used to determine whether a person chewed tobacco, smoked a pipe, rolled tobacco, or smoked cigarettes or cigars. Possible responses included: One of three options is available: 1) Yes, 2) No, or 3) Quit.

Drinking (alcohol) activities were determined by asking: “Did you drink any alcoholic beverages, such as beer, wine, or liquor in the past year, and if so, how often?”. Possible replies included: 1) Drink more than once a month; 2) Drink less than once a month, or 3) Do not drink.

**Depressive symptom manifestations**

Within the third experiment, depression-related symptoms were assessed using the 10-item CESD-10. With response possibilities ranging from 0 to 3 [0=Rarely or none of the time (less than 1 day); 1=Some or a little of the time (1-2 days); 2=Occasionally or a substantial amount of time (3-4 days); 3=Most or all of the time (5-7 days)], all 10 items originated from the initial 20-item CESD. With scores ranging from 0 to 30, participants were asked to rate “how often you felt this way during the past week.” The CESD-10 was created to identify experiences in populations associated with depression (Chen and Mui, 2014). A CESD-10 scoring of  ≥20 was stratified inside the depression group (D),  ≥10 for the depressive symptoms group (DS), and <10 for the group with no depressive symptoms (NDS) to examine influencing factors for depressive manifestations (Andresen et al., 1994).

**Statistical analyses**

Overall, 21.74% of the 21,100 sampled participants had low values and were excluded, leaving 16,512 records that were investigated through SPSS 26.0® (SPSS Inc.™, USA). Firstly, dataset vague descriptors were added. The categorical variable format is n (%). Secondly, a chi-square test was used to evaluate statistical variations across the NDS and combined groups (DS and D). Because age did not follow a normal distribution, ROC sensitivity analysis was performed to transform age into categorical variables. Thirdly, binary logistic regression analysis included all previously determined variables statistically significant through univariate analyses. In combination with formal determinations, a step-wise (forward-backward) protocol and maximum-likelihood estimation were employed, with the potential for forwarding/backward-stepping being 0.05 and 0.10, respectively. All p values were two-sided, and statistical significance was regarded to be conferred across all studies at p<0.05. The random forest (RF) algorithm (R package caret) was used to quantify the permutation importance of each testing dataset to deliver feature-importance analysis. The RF was a decision tree-based ensemble learning approach. The quantification of classifier performance loss after each prediction variable was randomly shifted was used to calculate the relevance of permutations. The eventual classifier performance loss can be measured using the area-under-the-curve (AUC), which measures how dependent the classifier is on each prediction variable because such shifts broke linkages between variables and intended outcomes. The 5-fold cross-validation (5 folds of training and testing with randomized data-split) was used to measure model performance reliably. The flowchart of this study was shown in **Figure 1**.

*Supplementary Section2: Sensitivity analysis of LR model and RF model.*


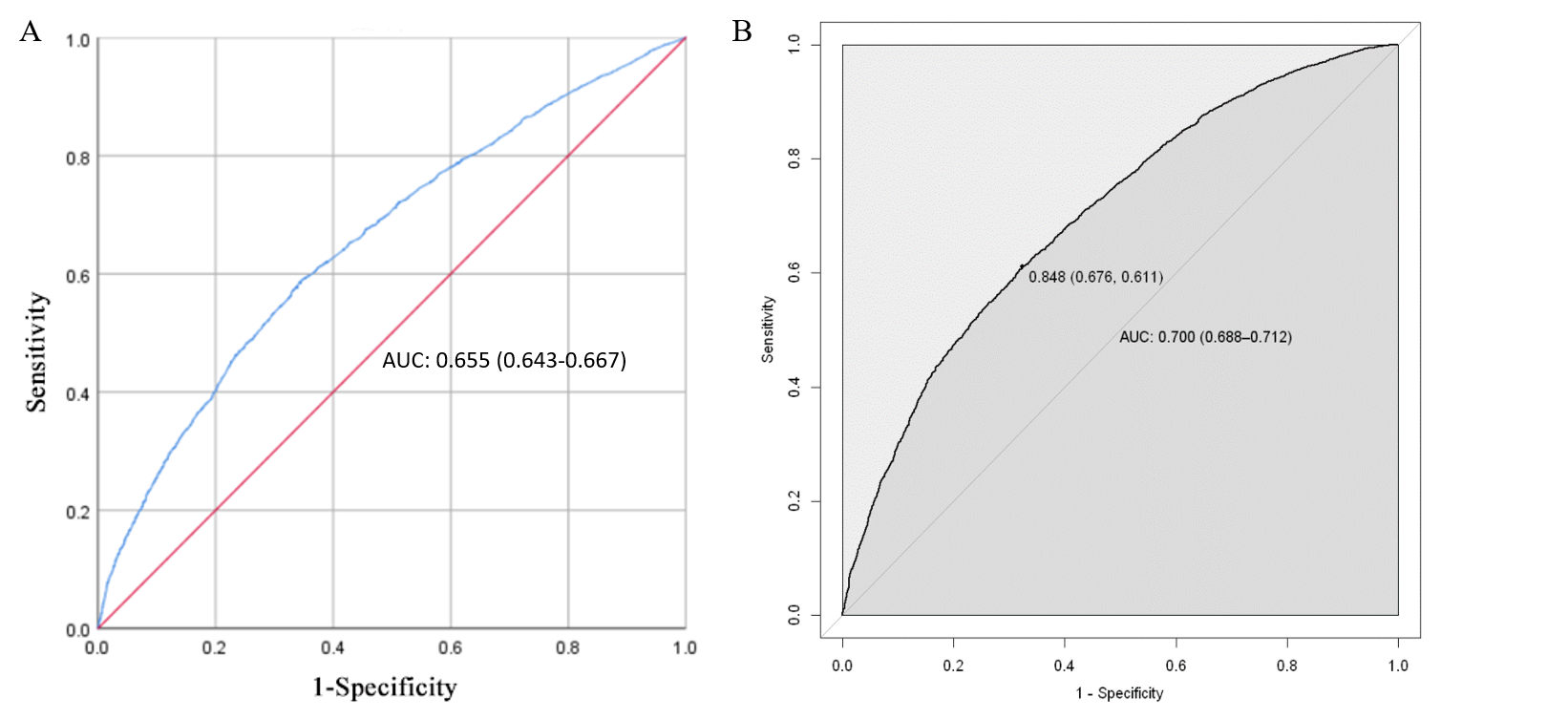


**Supplementary Figure 1**. Sensitivity analysis of LR model and RF model. (A) LR model; (B) RF model.

*Supplementary Section3:* *The weights of related factors of depressive symptoms.*

| **Supplementary Table 1.** The weights of related factors of depressive symptoms | |
| --- | --- |
| Variable | Weights |
| Chronic pains | 100.000 |
| Social Activity | 35.254 |
| Marital Status | 31.452 |
| Childhood Health Status | 29.714 |
| Loss all of Teeth | 29.328 |
| Gender | 28.524 |
| Frequency of Drinking | 27.524 |
| Smoking | 23.936 |
| Sporting Activities | 21.036 |
| State of Health | 20.130 |
| Changes in Health Status Following Last Interview | 19.934 |
| Location of Residential Address | 19.610 |
| ADL | 19.449 |
| Type of Residential Address | 19.062 |
| Born Address | 18.743 |
| Age Group | 18.727 |
